# Is there a relation between dental anxiety, fear and general psychological status?

**DOI:** 10.7717/peerj.2978

**Published:** 2017-02-15

**Authors:** Tuba Talo Yildirim, Serkan Dundar, Alihan Bozoglan, Tahir Karaman, Neval Dildes, Filiz Acun Kaya, Eyyup Altintas, Faruk Oztekin, Osman Atas, Hilal Alan

**Affiliations:** 1 Department of Periodontology, Firat University, Elazig, Turkey; 2 Department of Prosthodontics, Firat University, Elazig, Turkey; 3 Department of Orthodontics, Firat University, Elazig, Turkey; 4 Department of Periodontology, Dicle University, Diyarbakir, Turkey; 5 Department of Endodontics, Firat University, Elazig, Turkey; 6 Department of Pedodontics, Firat University, Elazig, Turkey; 7 Department of Oral and Maxillofacial Surgery, Inonu University, Malatya, Turkey

**Keywords:** Depression, Anxiety, Dental anxiety, Dental fear

## Abstract

**Background:**

Dental anxiety is a widespread problem in many populations. This problem can be a barrier to dental care and may lead to poor oral health. Dental anxiety may be related to psychological status.

**Aims:**

The aim of the present study was to assess the levels of dental anxiety, dental fear, Beck Depression, and state-trait anxiety according to age, gender and education level in patients at the periodontology clinic in the Diyarbakır Mouth and Dental Health Center.

**Study Design:**

A total of 231 patients (115 males, 116 females) filled out dental fear scale (DFS), dental anxiety scale (DAS), Beck Depression Inventory (BDI), state-trait anxiety inventory-state (STAI-S), and state-trait anxiety inventory–trait (STAI-T) questionnaires, and evaluations of DFS, DAS, BDI, STAI-S, and STAI-T were conducted according to age, gender, and education level.

**Results:**

The mean DFS, DAS, BDI, STAI-T, and STAI –S were 45.64, 9.15, 13.16, 38.90, and 40.18, respectively. There was a significant association among DFS, DAS, BDI, STAI-S, and STAI-T (*p* < 0.05). These surveys scores were significantly higher in females than males (*p* < 0.05). The results of this study indicated that gender age and education level have important effects on DFS, DAS, BDI, STAI-S, and STAI-T (*p* < 0.05).

**Conclusion:**

Dental anxiety and dental fear were found to be related to psychological status (BDI, STAI-S, and STAI-T) over time. There are some patients with unaddressed psychological distress.

## Introduction

Anxiety is characterized as an uncertain, unpleasant feeling accompanied by the premonition that something undesirable is about to happen (Marya et al., 2012). Dental anxiety is a widespread problem in populations of different countries (Edmunds & Buchanan, 2012; Newton et al., 2012; Teo et al., 1990; Tunc et al., 2005). Previous studies reported 64% of individuals nervous of dental treatment (Edmunds & Buchanan, 2012), with a smaller but still significant number of adults (2.4–3.7%) classed as dental phobics (Oosterink, De Jongh & Hoogstraten, 2009b; Stinson et al., 2007). Dental anxiety, which is important psychological problem, is a serious condition that also influences patients’ physical health (Kesim et al., 2012). Further, a practical dental procedure is a significant problem for many individuals with dental fear and anxiety. This problem can be a barrier to dental care, and the lack of proper care may lead to poor oral health (Chadwick, 2002; Edmunds & Buchanan, 2012; Newton et al., 2012). Treatment of patients with dental fear and anxiety is difficult for dentists. Dentists can become anxious when dealing with these patients because individuals with dental anxiety are more difficult to control, and, consequently, dental treatment procedures take a long time (Ilguy et al., 2005). In this respect, there is a compelling convergence of research suggesting that distressing or aversive experiences make people vulnerable and increase the risk of developing a broad range of anxiety disorders, such as general anxiety disorder (Oosterink, De Jongh & Aartman, 2009a).

The etiology of dental anxiety is various. Several genetic and environmental factors have been suggested, including congenital determinants, trauma, and past unfavorable dental experiences (Tunc et al., 2005). Locker, Shapiro & Liddell, (1996) suggested that dental anxiety was specifically related to invasive or painful treatment. De Jongh et al., (2006) found that highly anxious dental patients were about five times more likely to have ever experienced a horrific dental treatment in their life, and almost six times more likely to have ever experienced a violent crime than their low dentally anxious counterparts.

Dental anxiety is a complicated event that is affected by different variables (Klages et al., 2006). The level of dental anxiety may be affected by age, gender, and education level (Hagglin et al., 2000; Lopez-Jornet, Camacho-Alonso & Sanchez-Siles, 2014). In general women have more dental fear than men (Ragnarsson et al., 2003). Recent studies have shown that dental fear is more common among younger adults than older individuals (Pekkan, Kilicoglu & Hatipoglu, 2011). However, other studies have indicated that younger individuals (15- to 25-year-olds) have less dental fear than older individuals (Lahti et al., 2007). Also, severe dental fear is more common among patients with lower educational status or those who are single than among those with higher educational status and/or in a relationship (Hagglin et al., 2000). However, Lopez-Jornet, Camacho-Alonso & Sanchez-Siles (2014) reported that there was no relationship between the dental anxiety and various education levels.

Dental anxiety and fear could in itself be a psychological disorders. There are a lot of studies on this subject in literature. Further, previous studies reported that there is a relationship between dental anxiety and general fear and anxiety, as well as general psychological status (Fuentes, Gorenstein & Hu, 2009; Kesim et al., 2012). Although there are several studies based on dental fear and anxiety, there have not been enough studies which evaluate DFS, DAS, BDI, STAI-S and STAI_T together. Our study is different in that DFS, DAS and BDI, STAI-S, STAI-T were evaluated together according to age, gender and education level.

In the present study, our aim was firstly to assess the relation between dental anxiety, dental fear, and general psychological status; and secondly, to determine the dental anxiety, dental fear and general psychological status related to age, gender and education level.

## Materials and Methods

### Study population

This cross-sectional study was carried out at the Department of Periodontology in the Mouth and Health Center in Diyarbakır, Turkey. The study sample, which consisted of 231 patients (115 males and 116 females), was randomly drawn from the periodontology clinic. Three hundred patients were included in the study but only 231 patients completed questionnaires, 69 participant who have not complete all question of some tests not a few questions. Therefore, 69 participants could not be used due to missing data (less than 80% of the questions were answered) and these were excluded from subsequent data analyses such as previously studies (De Jongh et al., 2006; Gomez-de Diego et al., 2014; Oosterink, De Jongh & Aartman, 2008). These surveys were not given all the patients who applied to the clinic, only those who volunteered. Therefore, the number of participants was high.

A comprehensive definition of the sampling size had been previously published (Kanaffa-Kilijanska et al., 2014; Kesim et al., 2012). The project was approved by the Ethics Committee of Firat University (21.04.2015-08-10). At first, all participants were informed about this research both verbally and written. All patients volunteered to participate and written informed consent was taken from all the participants prior to the investigation. Then, the participants who agreed to participate in the study filled in the questionnaires. Inclusion criteria for the subjects in this study were as follows: participants who were at least 18 years old, with no cognitive impairments and eye diseases, and able to complete the questionnaire independently. Participants who were illiterate, noncooperative and who had a mental illness were excluded. Further, who had under the influence of an anxiolytic, sedative, or analgesic agent were given three days (for the passage the drugs) to complete the survey before they were excluded.

### Study design

Patients filled out DFS, DAS, STAI-S, and BDI to assess their dental fear, dental anxiety, general anxiety, and depression levels respectively. A week later, the same patients’ STAI-T scores were applied to determine their overall psychological status. Additionally, gender, age, and education level were noted by the researcher before patients filled out these surveys

### Measures

Dental fear levels were evaluated using DFS, as proposed by Kleinknecht in 1973. The DFS includes 20 items that assess issues related to avoidance treatment, physiological reactions, and stimuli associated with dental treatment. Dental anxiety was evaluated on a Likert scale of intensity that ranges from 1 (not afraid) to 5 (very afraid). Therefore, total scores range from 20 (not afraid) to 100 (terrified) (Kvale et al., 1997; Lopez-Jornet, Camacho-Alonso & Sanchez-Siles, 2014). The DFS used in this paper was the Turkish translation of the scale used by Kvale et al. (Firat, Tunc & Sar, 2006; Kvale et al., 1997).

Dental anxiety was evaluated by different questionnaires and scales (Hakeberg et al., 2001) such as the DAS proposed by Corah (1969). This scale measures the degree of anxiety related to dental treatment via a four item multiple choice questionnaire. Thus, total scores range from 4 (no anxiety) to 20 (high anxiety). DAS scores are classified in the following way: less than 12 indicates low anxiety, 12–14 indicates moderate anxiety, and scores greater than 14 indicate high dental anxiety. This scale is applied quickly (less than five minutes). Also, the reliability rate is high, and it has shown predictive accuracy (Ilguy et al., 2005).

Anxiety was assessed with the Spielberger STAI. This inventory includes two self-report scales. These self-report scales measure two different dimensions of anxiety: state anxiety and trait anxiety. The trait anxiety scale is used to describe how subjects generally feel (STAI-T), and the state anxiety scale reflects a response at a specific moment in time (STAI-S) (Albanidou-Farmaki et al., 2008). Possible scores vary from 20 to 80; the test scores of <30 may indicate little or no anxiety, scores 31–49 may indicate moderate anxiety, and scores higher than 50 indicate extreme anxiety or positive results of anxiety test (Lopez-Jornet, Camacho-Alonso & Sanchez-Siles, 2014). The STAI used in this paper was the Turkish versions (Mantar, Yemez & Alkin, 2010).

BDI was designed by Beck et al. (1961). It consists of 21 items which are presented in multiple-choice form and was designed to assess the severity of depression symptoms. Patient selected responses that best suited their current situation to determine the intensity/ severity of depression. It was adapted to Turkish by Hisli Şahin (1989). Scores range from 0 to 3 for each item. Thus, total scores range from 0 to 13 (minimal depression), 14–19 (mild depression), 20–28 (moderate depression), and 29–63 (severe depression). The scale’s minimum score is 0 and maximum score is 63 (Abolghasemi et al., 2015; Ayaz, Karanci & Aker, 2014).

### Statistical analysis

The SPSS 21 package program was used for statistical analysis. Variables were described as mean ± SD. The compliance of the data with the normal distribution probability/hypothesis was analyzed via the Kolmogorov–Smirnov test. In investigating the differences between parametric data, the analysis was performed by using the independent Student’s *t* -test in two group (gender) and ANOVA was applied and than differences between groups were evaluated by post-hoc Tukey test (age and education level). A Spearman correlation was done to assess the association between dental anxiety, dental fear, Beck Depression, state and trait anxiety. *p* < 0.05 were accepted as statistical significant.

## Results

The study sample consisted of 116 (50.2%) females and 115 (49.8%) males. Their mean age was 36.54 ± 13.34 years. In terms of education level, 12 participants had completed an elementary education, 100 had completed junior high school, 44 had completed high school, and 75 were university-educated.

The average DFS value of these patients was 45.64 ± 16.97. There was a statistically significant difference among education level and DFS (*p* < 0.05) (Table 1). The results showed that gender and age had a significant effect on dental fear (*p* < 0.05) (Tables 2 and 3).

**Table 1:** Relationship between education and DFS, DAS, BDI, STAI-T, STAI-S level of patients.

Education level	DFS	DAS	BDI	STAI-T	STAI-S	N
Primary school	77.66 ± 6.87^a^	14.91 ± 2.57^a^	34.83 ± 10.76^a^	55.16 ± 5.62^a^	54.91 ± 4.10^a^	12
Secondary school	64.18 ± 10.67^b^	12.63 ± 2.30^b^	24.11 ± 11.05^b^	50.15 ± 8.16^b^	50.65 ± 7.65^b^	44
High school	43.52 ± 11.96^c^	8.70 ± 2.31^c^	11.01 ± 8.68^c^	36.97 ± 9.30^c^	38.97 ± 9.49^c^	100
University	32.38 ± 8.80^d^	6.87 ± 1.93^d^	6.16 ± 5.01^d^	32.26 ± 7.80^d^	33.30 ± 7.14^d^	75

**Notes.**

* Within the same measurement category, values with the same capital letter are not statistically significant by Tukey’s method for post hoc analysis.

**Table 2:** Relationship between gender and DFS, DAS, BDI, STAI-T, STAI-S level of patients.

Gender	DFS	DAS	BDI	STAI-T	STAI-S	N
Male	37.90	7.65	9.15	35.38	37.26	115
Female	53.25	10.63	17.14	42.38	43.08	116
	^*^p < **0.05**	^*^p < **0.05**	^*^p < **0.05**	^*^p < **0.05**	^*^p < **0.05**	

**Notes.**

* Student’s *t* test *p* < 0.05.

**Table 3:** Relationship between age group and DFS, DAS, BDI, STAI-T, STAI-S level of patients.

Age	DFS	DAS	BDI	STAI-T	STAI-S	N
10–19	58.45^a^	11.83^a^	23.16^a^	46.45^a^	46.33^a^	24
20–29	52.77^b^	10.53^b^	16.63^b^	42.74^b^	45.00^b^	63
30–39	45.02^c^	9.14^c^	13.14^c^	38.65^c^	40.22^c^	49
40–49	39.91^d^	7.66^d^	10.02^d^	36.66^d^	37.5^d^	45
50–59	35.20^e^	7.20^e^	5.74^e^	32.06^e^	33.16^e^	43
60–69	41.85^f^	9^f^	13.71^f^	36.42^f^	35.85^f^	7

**Notes.**

* Within the same measurement category, values with the same capital letter are not statistically different by Tukey’s post hoc analysis.

The mean DAS score for the sample was 9.15 ± 3.2. There was a statistically significant difference among education level and DAS (*p* < 0.05) (Table 1). Women had significantly higher scores than men (*p* < 0.05) (Table 2). There was a significant association between age and dental anxiety (*p* < 0.05 ) (Table 3).

The mean BDI score was 13.16 ± 11.59; the mean STAI-S score was 38.90 ± 11.17, and the mean STAI-T score 40.18 ± 10.74. There was a statistically significant difference among education level and BDI, STAI-S, STAI-T (*p* < 0.05) (Table 1). These results were significantly higher in females than in males (*p* < 0.05) (Table 2). There was a statistically significant association among age and BDI, STAI-S, and STAI-T (*p* < 0.05) (Table 3). The means and standard error of dental fear, dental anxiety, BDI, STAI-S, STAI-T for the total population by age are shown in Table 3. Also, a statistically significant association was found among DFS, DAS, BDI, STAI-S, and STAI-T (*p* < 0.05).

## Discussion

In spite of the technological advancements in modern dentistry, anxiety and fear associated with dental treatment is very common (Bedi & McGrath, 2000). A recent study showed that 73%–79% of individuals have experienced at least some anxiety during dental treatment (Marya et al., 2012) This study aimed to investigate the relation between dental fear, dental anxiety, state-trait anxiety, and depression (as measured by the Beck Depression Inventory) according to age, gender and education level.

What was shown by the results of this study is that dental anxiety is more common in women than men, and women have a high level of dental anxiety. Consistent with the results in this study, Fallea, Zuccarello & Cali (2016), reported that the prevalence of dental fear is significantly higher in females. Previous investigations focusing on the frequency of dental fear and anxiety and their correlation with gender are confirm our results (Carlsson, Hakeberg & Wide Boman, 2015; Tunc et al., 2005). Physiological sensations related to stress, depression, fear, social phobia, and panic are widespread in women, and these may be related to dental anxiety (Locker, Poulton & Thomson, 2001). According to this data, it can be said that women are affected by more negatively sensational status than men and that anxiety is positively related to psychological status. The findings of this study proved similar to those found in previous studies that reported women tend to be more anxious than men and showed higher DAS scores (Stabholz & Peretz, 1999).

Adults with higher educational levels are generally more aware of existing medical services, and as such access information about dental care and dental practices (Pakpour et al., 2014). Furthermore, evidence suggests that educational status is related to the capacity of dental awarenes and regular dental appointment attendance (Godin et al., 2010; Pakpour et al., 2014). When education level increases, the dental awarenes increases so dental fear and anxiety levels decrease (Yildirim, 2016). Previous studies identified education level as a factor that affects the dental anxiety level of patients (Ekanayake & Dharmawardena, 2003; Teo et al., 1990). For example, Hallstrom and Haling reported that the prevalence of dental phobia was higher in patients with a low education level (Hallstrom & Halling, 1984). The results of this study showed that a relationship exists between dental anxiety and different education levels. In the present study, patients with a primary school education had the highest anxiety scores and therefore were more anxious about dental treatment compared with those with secondary school, high school, university, and postgraduate educations. Similar to this study, Ilguy et al. (2005) reported that education level is imported factor to assess dental fear and anxiety. In contrast, other studies reported that higher educational levels were associated with higher dental anxiety (Erten, Akarslan & Bodrumlu, 2006; Teo et al., 1990), and some researchers found that individuals with both low socioeconomic status and low education level have more anxiety (Yildirim, 2016).

Specific researchers who conducted these studies reported that there is a relationship between dental anxiety and general fears and anxiety, as well as general psychological status (Abrahamsson, Berggren & Carlsson, 2000; Frazer & Hampson, 1988). An important result of the present study was the discovery of a significant relationship between dental anxiety, dental fear, trait and state anxiety, and depression (as measured by the BDI). Our study showed that patients with high dental anxiety and fear tend to present high trait-state anxiety. Several reports are in agreement with our findings (Lago-Mendez et al., 2006; Weisenberg, Kreindler & Schachat, 1974). Locker and Fuentes reported that there is a relationship between dental anxiety and psychological status (Locker, Poulton & Thomson, 2001). Mendez evaluated individuals with dental anxiety who were consulting for third molar removal for a possible association with general trait anxiety and reported that trait anxiety showed a significant positive relation with both DAS and DFS scores (Lago-Mendez et al., 2006).

Dental fear and anxiety were found to be associated with generalized anxiety disorder and depressive disorders, even when the most significant confounding factors were controlled for (Locker, Poulton & Thomson, 2001). For the purposes of our study, we used BDI, a test that measures depression. The results of this study indicated that patients who have both anxiety and depressive disorders are more likely to have high levels of dental fear than patients with either anxiety or depressive disorder, or those without these disorders. Locker, Poulton & Thomson (2001) confirmed these results. Pekkan, Kilicoglu & Hatipoglu (2011) reported that dental anxiety is positively correlated with depressive disorders. Also, similar to our study, Halonen et al. (2014) reported that dental anxiety is related to general anxiety in both women and men.

In the present study we did not evaluate only the relationship between DAS or DFS and BDI or STAI just like the previous studies. Three different indexes (BDI, STAI-T, STAI-S) showing general psychological status were used and their relationship with DAS and DFS were evaluated. Thus, far more comprehensive results were contributed to the literature than in previous studies. At the same time, a different analysis was conducted for the gender, age and education, so also additional results were contributed to other studies examining the general psychological status.

## Conclusion

Dental fear is a widespread problem both for dentist and patient. Within the limitation that all participants selected only one center, the study indicated that women, young people, and those who have a low level of education have high levels of psychological disorder. In addition, these individuals have high levels of dental anxiety and dental fear, and there is an association between psychological disorders and dental anxiety, and dental fear over time. In this study, we measured anxiety and depression related to dentistry, and in the future the study would need to be repeated with a non-dentistry sample. There are some patients with unaddressed psychological distress.

##  Supplemental Information

10.7717/peerj.2978/supp-1 Data S1 Responses of participants

## Additional Information and Declarations

### Data Availability

The following information was supplied regarding data availability: The raw data has been supplied as Data S1.
